# A case report of the sustained and rapid response of bevacizumab in a TP53-positive breast cancer and liver metastatic patient through personalized medicine

**DOI:** 10.3389/fonc.2022.940678

**Published:** 2022-09-02

**Authors:** Mohammad Reza Eskandarion, Zahra Tizmaghz, Bahram Andalib, Nasser Parsa, Seyed Amir Hossein Emami, Reza Shahsiah, Mohammad Ali Oghabian, Reza Shirkoohi

**Affiliations:** ^1^ Cancer Research Center, Cancer Institute, Imam Khomeini Hospital Complex, Tehran University of Medical Sciences, Tehran, Iran; ^2^ BESTforPM (Biomarker Evaluation and Supervision Team for Personalized Medicine), Imam Khomeini Hospital Complex, Tehran University of Medical Sciences, Tehran, Iran; ^3^ Department of Radiation Oncology, Cancer Institute, Imam Khomeini Hospital Complex, Tehran University of Medical Sciences, Tehran, Iran; ^4^ Radiation Oncology Central Clinic of Karaj (ROCK), Karaj, Iran; ^5^ Iranian Cancer Association, Tehran, Iran; ^6^ Department of Hematology and Medical Oncology, Imam Khomeini Hospital Complex, Tehran University of Medical Sciences, Tehran, Iran; ^7^ Department of Anatomical and Clinical Pathology, Cancer Institute, Imam Khomeini Hospital Complex, Tehran University of Medical Sciences, Tehran, Iran; ^8^ Medical Physics Department, Faculty of Medicine, Tehran University of Medical Sciences, Tehran, Iran

**Keywords:** breast cancer, liver metastasis, tumor markers, target therapy, personalized medicine, NGS

## Abstract

HER2-positive metastatic breast cancer is much less frequent than other subgroups of breast cancer. Treatment options for this cancer are mostly limited to systemic chemotherapy, which leads to moderate improvements. Targeted therapy against malignant breast cancer requires the identification of reliable biomarkers for personalized medicine to obtain the maximum benefit of this therapy. Any mutations in the TP53 signaling pathway can be considered as a significant causative factor of breast cancer, for which the identification of target genes plays an important role in selecting the appropriate treatment. The use of personalized gene expression profiling could be valuable to find the direct target of the treatment in this case. The present study assessed the genetic profile of an HER2-positive metastatic breast cancer patient (with a liver metastasis) and figured out a complete and sustained response to bevacizumab. According to the results of next-generation sequencing (NGS) analysis, the patient’s genetic profile showed an increased expression of p4EBP1 and PTEN and the activation of the mTOR signaling pathway with a mutation in the TP53 gene. Based on the common treatment of similar profiling, we administrated bevacizumab/Taxol/Gemzar chemotherapy up to six courses. Accordingly, as the response to treatment was revealed by reducing the volume of the liver metastasis from 4 to 1.4 cm, metastasectomy was performed as a complementary treatment. Hence, personalized gene expression profiling not only is useful for targeted therapy but also could be recommended to avoid prescription of non-responsive drugs.

## Introduction

Nowadays, personalized medicine has made it possible to diagnose, treat, or prevent a specific disease using the genetic structure of each patient. The ultimate goal of personalized medicine is to provide a set of markers that can be used to assess the risk of developing a disease throughout the life of each individual in the presence of environmental variables (1).

Most cancer cells do not respond well to conventional therapies, and there is still a risk of recurrence, indicating very high heterogeneity of the disease and the involvement of particular molecular mechanisms specific to each tumor. Therefore, each individual shows an exclusive response to treatment based on a specific genotype (2).

Breast cancer is a highly heterogeneous disease caused by genetic and epigenetic mutations and is one of the most important causes of cancer mortality (3). Different types of breast cancer including ductal carcinoma *in situ* (DCIS) and invasive lobular carcinoma (ILC) have been reported in terms of their clinical features (4). The most common types are invasive ductal carcinoma (IDC) and ILC. IDC makes up about 70%–80% of all breast cancers (4). As patients differently respond to each treatment, the molecular analysis as well as the clinical and pathological findings play an important role in the targeted therapy considering the advances in the genomic field (5).

One of the remarkable targets in cancer signaling pathway is tumor protein P53 (TP53) protein, which is a multifunctional transcription factor in the wild type and is involved in cell-cycle control, DNA maintenance, genome integrity, post-DNA damage repair, and apoptosis (6). The loss of TP53 function results in the replication of damaged DNA. Therefore, tumors with activated TP53 mutations are expected to have more mutations in other genes that lead to an increase or decrease in the expression of other genes (7).

TP53 is a multifunctional tumor-suppressor gene that is often active in neo-vascularization following a number of inhibitory mechanisms (8). For instance, TP53 promotes the degradation of the hypoxia-inducible factor 1 (HIF-1) in the cell. In reaction to oxygen deficiency, HIF-1 functions as a primary transcriptional activator of vascular endothelial growth factor-(VEGF-)mediated angiogenesis (9). Although the interaction between TP53 and HIF-1 is not well understood, TP53 tends to influence angiogenesis *via* other pathways. As compared with non-overexpressing tumors, the overexpression of VEGF in breast cancer patients contributes to shorter relapse-free and total survival periods (9).

Many patients with various cancers are referred to the Biomarker Evaluation & Supervision Team for Personalized Medicine (BESTforPM), Imam Khomeini Hospital Complex, Tehran University of Medical Sciences, Tehran, Iran. These patients usually suffer complicated, metastatic, or end-stage cancers, most of which are resistant to conventional chemotherapy methods. Therefore, patients’ genetic profiles are usually checked with the development of personalized medicine based on the decision of a multidisciplinary team for some patients. The results of personal medical tests would lead to the presentation of a better treatment for those patients. In the present study, we described the case of a patient with phenotypic, metastatic breast cancer that unpredictably reacted to bevacizumab therapy. A single reportable somatic TP53 mutation was discovered during genomic profiling of her metastatic liver specimen. The available literature suggests conflicting evidence that supports the role of this mutation in cancer (3). Some evidence proposes that it may stimulate the angiogenesis signaling pathway (7). The reported case emphasizes the importance of further research examining the function of TP53 mutations in cancer development. Moreover, therapeutic response also highlights that personalized medicine, unlike traditional chemotherapy methods, can contribute to targeted therapy.

## Materials and methods

### Clinical presentation and family history

The patient was a 48-year-old woman at premenopausal stage. In November 2014, she was diagnosed with IDC, grade II, T1c N1 M0, and ER+/PR+ HER-2 weakly (+2) positive. There was no history of breast cancer and no proof of BRCA1/2 mutations among the patient’s relatives. In December 2014, after the initial diagnosis of ductal carcinoma, the left breast underwent lumpectomy, and then chemotherapy was given by doxorubicin, cyclophosphamide (four courses), and Taxol (four courses). The treatment process was kept going on with radiotherapy (50 Gy/25Fr + tumor bed electron boost 10 Gy/5Fr in 30 sessions) from May 2015 to July 2015. Following the chemotherapy, she received 17 courses of Herceptin (440 mg every 3 weeks).

**Figure 1:**
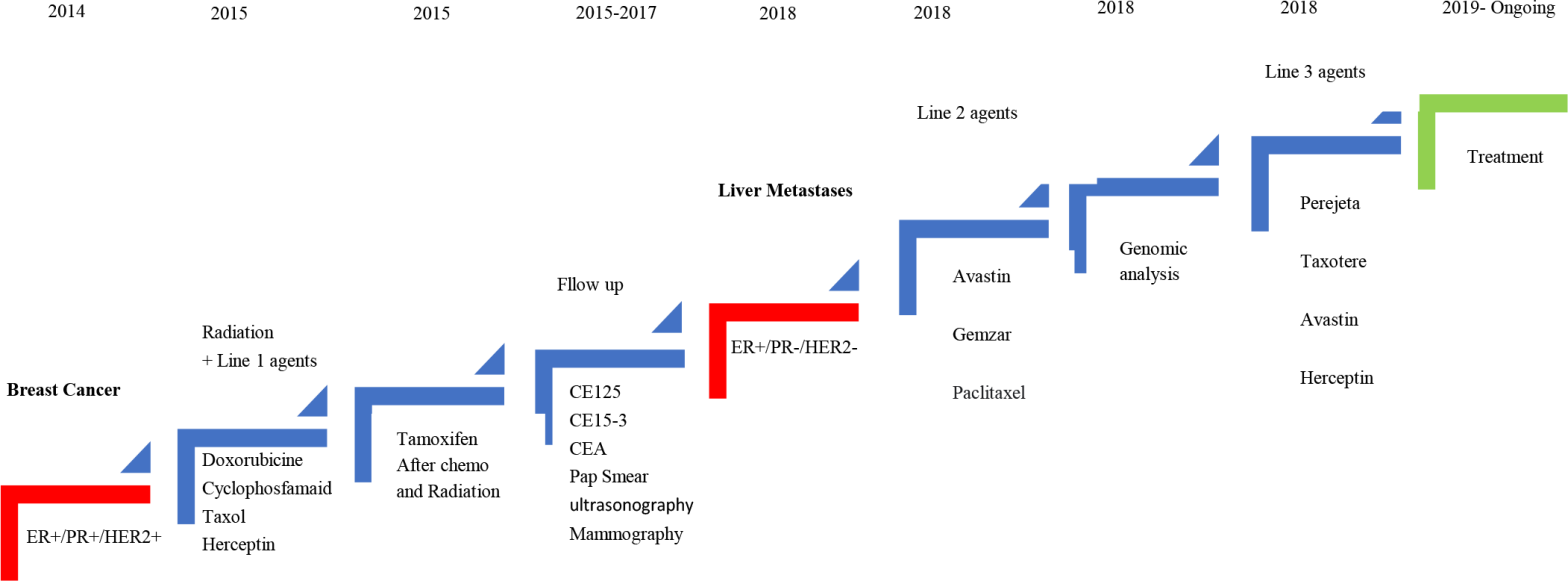
Schematic diagram of the patient’s clinical course indicating treatment, duration, and disease recurrence.

Hormone therapy started by tamoxifen for 8 months from June 2015 to February 2016; however, due to some side effects it was switched to letrozole which was consumed for about 2.5 years from February 2016 to July 2018 (**Figure 1**). During the first 2 years, diagnostic follow-up was performed every 3 months, and then every 6 months by evaluating tumor markers using blood tests (CA 125, CA15-3, and CEA), the results of which were normal throughout the mentioned period.

In addition, other follow-up diagnostic modalities for this patient included breast ultrasound twice a year as well as the annual mammography, which indicated no evidence of recurrence or metastasis, other than a suspicious lesion located at the surgical incision site, which was reported normal by MR mammography. Furthermore, Pap smear tests and clinical gynecology investigations performed every 6 months were reported to be normal.

**Figure 2:**
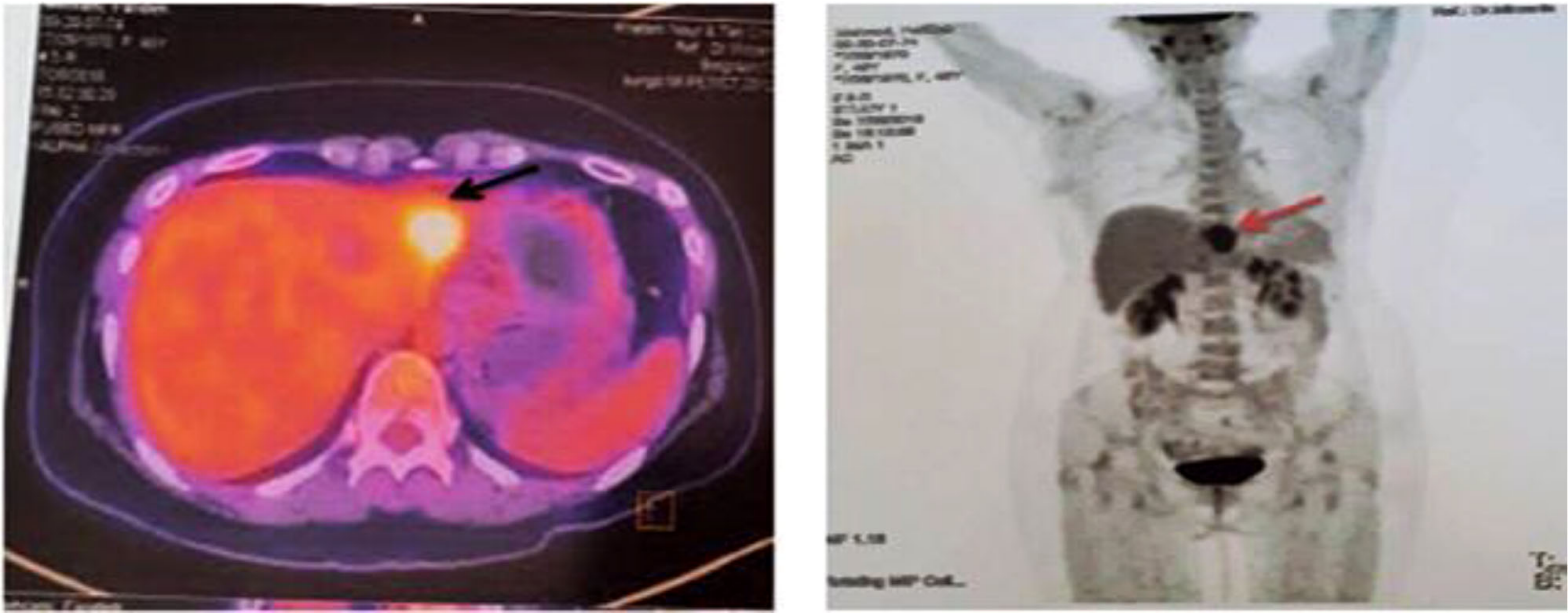
First PET/CT on 26 July 2018. The arrows show a fluorodeoxyglucose (FDG) avidity-standardized uptake value (SUVmax = 9.2) hypo-attenuating lesion with partial necrosis with the left liver lobe, compatible with metastatic disease.

In July 2018, without any symptoms and only based on the patient’s request for abdominal and pelvic ultrasonography, a hypodense mass with approximate dimensions of 32 × 41 mm was reported in the left liver lobe, and soon after a liver metastasis was confirmed by performing biopsy (**Figure 2**).

A positron emission tomography (PET) scan was performed. With the exception of the mentioned liver mass, the reports of other sites and phasic lung scan showed normal results. The patient was presented at the liver tumor board. Liver metastasectomy indications were discussed and recommended as a treatment modality along with the systemic therapy. Due to the limitations of approved common chemotherapy for breast cancer and the probability of developing chemoresistance, it was suggested to examine the patient in terms of genetic by NGS (the Ion Torrent Genexus System Platform) and tumor characteristics as well as the pattern of biomarkers to arrive at the best decision to choose effective systemic therapy agents including targeted therapy and find out clinical benefits or lack of benefits about each systemic therapy agent in order to raise the chance of treatment response before the golden time expiration.

## Results

### Genomic analysis

Among the 315 cancer-related genes studied, genomic profiling of the liver specimen in January 2019 reported a single reportable mutation: TP53 (**Table 1** and **Supplemental Table 1**).

**Table 1:** A summary of the results of the patient’s protein expression.

TP53	PTEN	TS	CD8	PosphoRb	p4EBP1	PD-L1	MSI	TMB	PIK3CA	BRCA1/2
**+**	+	+	+	-	**↑**	**↓**	No	No	WT	WT

+, Positive Expression.

-, Negative Expression.

↑, High Expression.

↓, Low Expression.

WT, Wild Type.

Mechanistic target of rapamycin (mTOR) activation was shown with a positive phosphorylation of 4EBP1 (p4EBP1) expression (an mTOR c1 effector) and phosphatase and tensin homologue (PTEN) positivity. In addition, PTEN was present to block the phosphatidylinositol-3-kinase (PI3K) pathway and fulfilled its function as a tumor suppressor, preventing cancer cell proliferation. Furthermore, NGS data (the Ion Torrent Genexus System Platform) revealed that this patient had wild-type PIK3CA. Overall, these data indicated that PI3K and dual PIK3/mTOR inhibitors were not correlated with therapeutic benefits in this patient. The immunogram test showed a poor propensity for immunotherapy. Despite the existence of CD8+ T cells in the tumor, we observed that PD-L1 expression was poor. Furthermore, no microsatellite instability (MSI), tumor mutational burden (TMB), or sensibility/resistance mutations were found. Preexisting CD8+ T cells, which were distinctly located at the invading tumor margin, were linked to PD-1/PD-L1 immune inhibitory axis expression and might predict therapy response. Furthermore, an improvement in CD8+ T cells in serial tumor samples was associated with a stronger response during treatment. However, since CD8 expression was impaired by tumor heterogeneity and temporal variability, measuring CD8+ T-cell infiltration alone did not have a strong predictive value. As a result, we could not rule out the possibility of a boost from immunotherapy care centered on the optimistic CD8 lymphocyte infiltration. The tumor ID profile in biopsy showed a single reportable somatic mutation: TP53-cDNA: c.584T>C. AA: p.I195T. VarFreq: 35%.

### Treatment outcomes

According to the results of the genetic profile report, chemotherapy with bevacizumab, Taxol, and Gemzar was continued for up to six courses. The response to treatment was achieved with a reduction of liver metastasis from 4 to 1.4 cm. Subsequently, the patient was referred for liver metastasectomy, which was performed first through laparoscopy procedure and then through liver metastasectomy as an open surgery.

**Figure 3:**
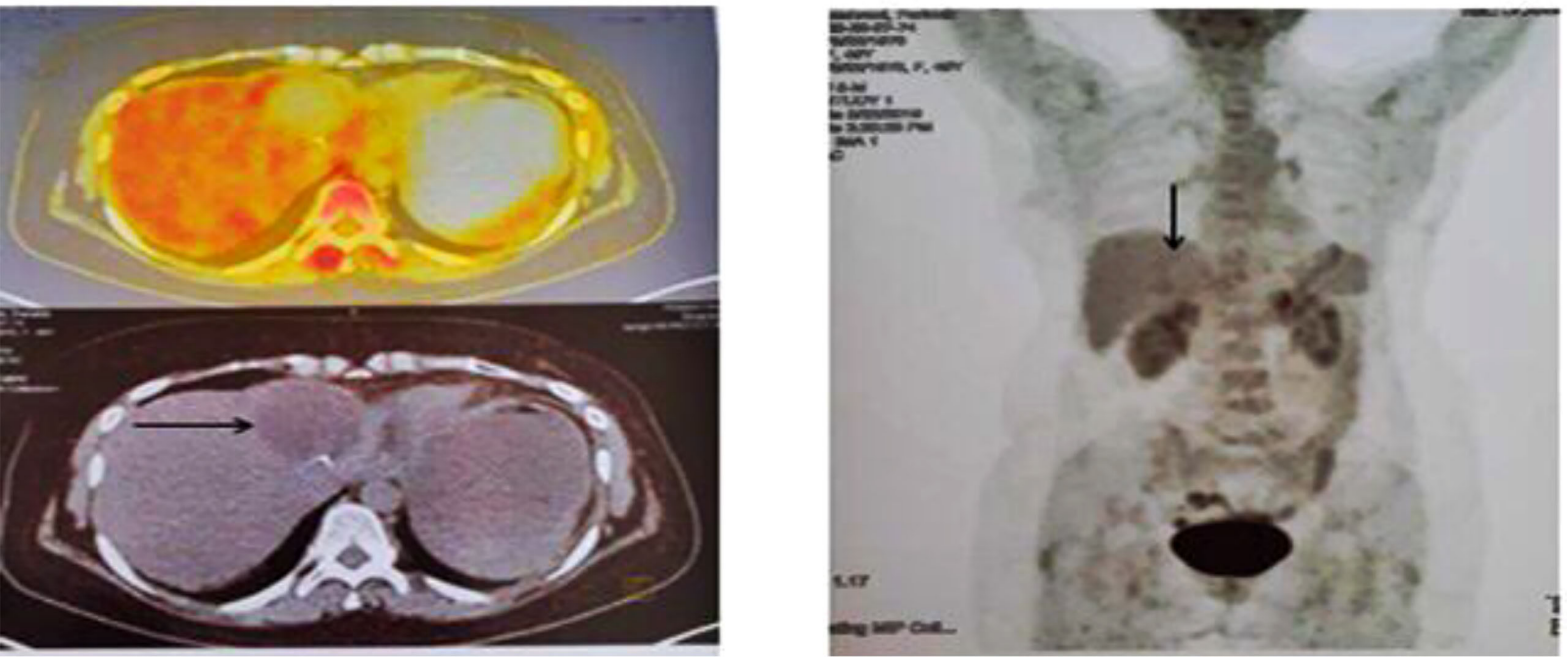
Second PET/CT on 23 May 2019. When compared with prior FDG PET/CT scan, the patient status post left liver lobe segment 4 resection, with non-FDG-avid post-surgical change in the surgical bed, which was compatible with an excellent complete metabolic response to therapy.

**Figure 4:**
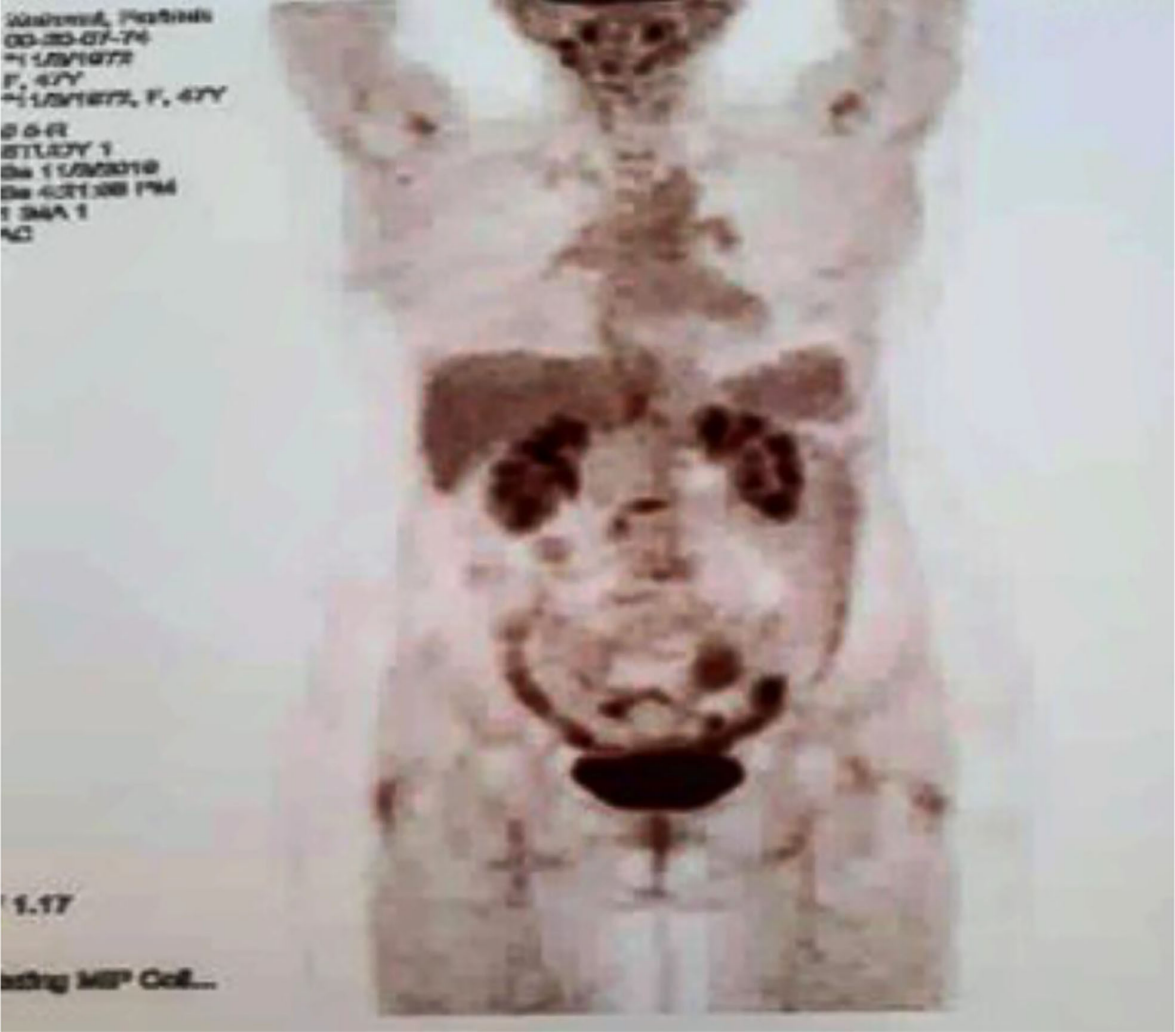
Third PET/CT on 03 November 2019. No metabolic evidence of malignancy in the images of the target parts of the body, which was compatible with continued remission.

At the end of the third line of treatment with Herceptin, Perjeta-Taxotere was started in March 2019. The reports of the PET scan provided in May 2019 and November 2019 indicated a complete response to treatment (**Figures 3**, **4**). Moreover, the diagnostic follow-up demonstrated the patient’s normal status currently.

## Discussion

**Figure 5:**
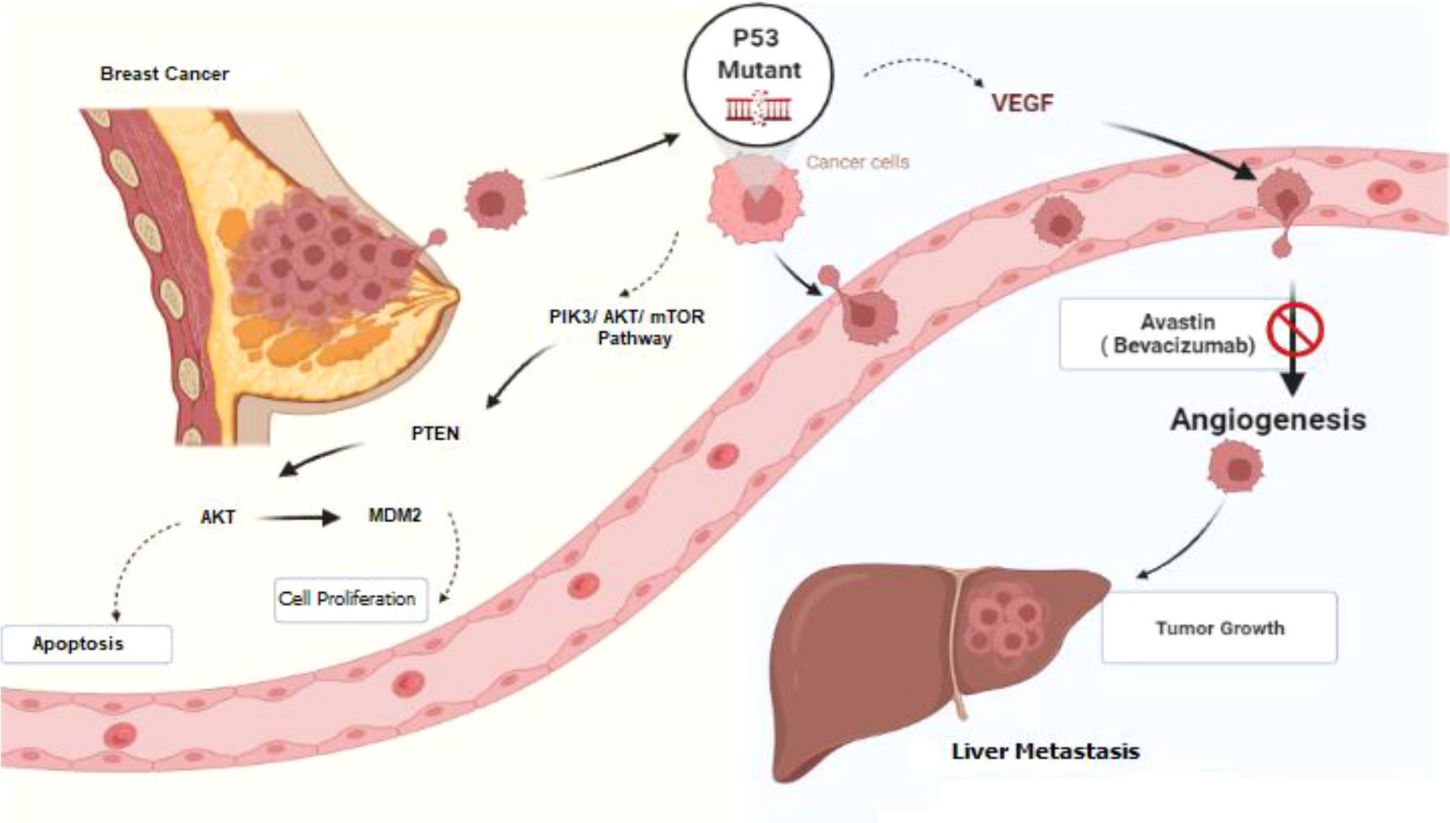
Schematic diagram of the signaling pathway from breast cancer to liver metastasis. TP53 protein is one of the important targets in the cancer signaling pathway and is involved in cell-cycle control, apoptosis, and angiogenesis. Bevacizumab is a VEGF-A monoclonal antibody against circulating VEGF and can inhibit tumor angiogenesis. This figure is created by Biorender.com.

Despite their high occurrence in cancer, there are currently no approved targeted therapies for TP53 mutations. According to a newly released retrospective review, patients with advanced cancers harboring a TP53 mutation have a longer progression-free survival on drug regimens containing bevacizumab (Avastin®; Roche, Basel, Switzerland) (**Figure 5**) (7).

In the current study, the patient treatment was initiated in 2014 with conventional chemotherapy based on standard guidelines, and all treatment policies including surgery, chemotherapy, radiotherapy, hormone therapy, and patient follow-up were performed. In 2018, a liver metastasis was observed as a result of diagnostic follow-up performed due to the patient’s request and without any specific symptoms or predicted risk factors. However, the multidisciplinary team consultation, oncologist–geneticist cooperation, and joint clinical decision making were benefited from in the second line of treatment, and the patient’s treatment plan was determined considering the personalized medicine and genetic analysis in order to find out the best treatment approach. As explained above, based on NGS findings, bevacizumab was determined as the first and best recommended choice of treatment and led to the dramatic response of the liver metastasis and prolonged the patient’s disease-free and overall survival rate. However, based on standard guidelines, bevacizumab is rarely employed in the therapeutic plan of patients with breast cancer and a liver metastasis and is more preferred in colorectal metastases and brain tumors. Therefore, it seems that if personalized medicine, rather than the conventional chemotherapy methods, was used from the very beginning of the treatment, a liver metastasis might not have occurred for this patient.

The valuable finding of this study is that in addition to the benefits of the personalized medicine and the corresponding treatment decision along with tumor identification, we also figured out the patient’s resistance to a specific part of the first line of chemotherapy, which suggested that the main agent in her previous chemotherapy was not effective. It should be mentioned that the instructions provided in the guidelines always consider the personalized medicine for the second line of treatment as an inclusion criterion. If we had the option to apply the personalized medicine for this patient immediately after the initial diagnosis, it would have helped us to choose more effective chemotherapy agents or change the regimens in order to improve the treatment in the first line. Furthermore, by doing so, the prescription of ineffective drugs with their side effects would have been avoided.

However, very few studies have been published on the use of bevacizumab in the treatment of metastatic breast cancer along with a liver metastasis. In line with the present study, Ogata et al. (10) used the combination of bevacizumab/paclitaxel/carboplatin to treat the liver metastasis in triple-negative breast cancer with mutations in the BRCA2 gene.

Huemer et al.’s study also reported about a woman with HER2-positive breast cancer that was highly symptomatic because of extensive pulmonary involvement. They used the combination of docetaxel/trastuzumab/bevacizumab that led to a significant response. The mentioned study revealed a significant association between treatment success and the addition of anti-VEGF antibody. The employed treatment was in agreement with the current study (11).

The role of bevacizumab in breast cancer is still controversial. Several studies demonstrated an improvement in terms of progression-free survival when bevacizumab was added to standard chemotherapy. However, on the other hand, the toxicity rate was increased and no benefit was observed in terms of the overall survival rate (12, 13). In line with numerous previous studies that have used surgery to treat liver metastasis caused by breast cancer, the present study also employed surgery in addition to the targeted therapy (14, 15).

The majority of p53 mutations have been found in the core hot spots of the DNA-binding domain (16). The current result of tumor profiling on biopsy showed a somatic mutation in TP53-cDNA: c.584T>C. AA: p.I195T which was found in the protein’s DNA-binding domain. This mutation was described as highly destabilized and linked to poor DNA-binding affinity (17). The variations found in TP53 in advanced cancers were scattered over multiple exons encoding the gene, suggesting that they were normal variations in exon 5 (n = 23. 33.3%), followed by exon 6 (n = 14, 20.3%), exon 7 (n = 12 17.4%), exon 4 (n = 9, 13.0%), exon 8 (n = 9 13.0%), and exon 9 (n = 2, 3.0%). More studies are required to specify which causes such as tissue of origin or a rare TP53 mutation influence bevacizumab responsiveness (7).

Several studies have been performed to examine the effect of somatic TP53 mutation in various cancers. One study in this respect showed a mutation in TP53 and its association with the increased expression of VEGF-A as the major ligand in the VEGF/VEGFR pathway, which can play a key role in regulating angiogenesis (18). The presented results refer to previous preclinical evidence, which connected TP53 to an increased VEGF expression and vessel density in head and neck tumors (18), breast carcinoma (19, 20), and bone marrow stromal cells in leukemia patients (21). Furthermore, the p53 protein controls angiogenesis, at least in part, by binding directly to the HIF- subunit and inducing HIF- degradation (9). Bevacizumab, as a VEGF-A monoclonal antibody against circulating VEGF, is one of the most common oncology medications. It inhibits tumor angiogenesis by blocking this ligand from communicating with its receptor (9, 22). This medication is licensed for implementation in a number of cancers including kidney cancer, colon cancer, non-small cell lung cancer (NSCLC), and glioblastoma. The effect of bevacizumab on survival in non-selected patients is limited, and the FDA revoked its approval in metastatic breast cancer in 2011 (23), although the National Comprehensive Cancer Network^®^ (NCCN^®^) Clinical Practice Guidelines in Oncology for Breast Cancer define the recommendation as follows: “Bevacizumab in combination with Paclitaxel is an appropriate therapeutic option for metastatic breast cancer” (24). In phase II clinical trials in metastatic breast cancer, bevacizumab showed positive efficacy and was well tolerated as a single agent. In phase III trials, bevacizumab with capecitabine, as compared with capecitabine alone, had little advantage in the progression-free survival, which may be attributed to the patient population, advanced stage at diagnosis, weak prognostic indicators, and also the employed chemotherapy protocol. Because of the role of other proangiogenic factors in breast cancer development and the variability of VEGF expression in breast cancer, a more targeted approach to anti-VEGF therapy may be advantageous (25). The association between TP53 mutations and positive results found in clinical trials of patients treated with anti-angiogenesis agents may be explained by attending to the relationship between TP53 mutations and the VEGF-A expression (7). Although previously published data on the predictive value of VEGF-A circulation have not been shown to correlate with patient recovery after bevacizumab treatment, it is also known that blood-derived VEGF-A levels are not well correlated with the VEGF-A tissue expression (25).

Other studies have also revealed that the p53 protein inhibits angiogenesis and neo-vascularization (8, 26). In addition, numerous tumor cell lines and xenograft tumor models have shown a connection between the p53 mutation and neo-vascularization (7). Scientific evidence indicated that p53 mutational status was not correlated with survival in patients with colorectal cancer treated with bevacizumab (27). As the pattern of cancer treatment changes from chemotherapy regimens to personalized medicine, the discovery of reliable biomarkers for treating cancer patients by targeted therapies is essential. Breast cancer treatment has so far been accepted using targeted HER2 therapies for HER2-positive and anti-hormonal therapies for ER +/PR + (28).

The basis of this type of treatment is the inhibition of growth factor receptors, inhibition of angiogenesis, and induction of apoptosis in tumor cells, which can be achieved using monoclonal antibodies and small-molecule inhibitors (29).

However, in order to extend this strategy, researchers will continue to specify biomarkers predicting therapeutic responses. Anti-angiogenic therapies (drugs targeting the neo-vascularization mechanism that helps tumors to self-sustain) will profit from the development of a new biomarker in particular.

## Conclusion

The present study presented an identified case of breast cancer with a phenotype that caused several chemotherapy courses to fail. Therefore, cancer gene expression profiling was used to amend the treatment based on her genetic tumor information. Following the detection of a change in TP53, it was decided to add targeted therapy with bevacizumab, as a result of which the patient experienced an exceptional 6-month response to the bevacizumab regimen. It can be concluded that recommendation of personalized gene expression profiling from the beginning in guidelines could be beneficial to find specific targets and prevent the prescription of non-responsive drugs.

## Data availability statement

The original contributions presented in the study are included in the article/**Supplementary Material**. Further inquiries can be directed to the corresponding authors.

## Ethics statement

Ethical review and approval was not required for the study on human participants in accordance with the local legislation and institutional requirements. The patients/participants provided their written informed consent to participate in this study.

## Author contributions

We are a team of multi-disciplinary known as experts BEST for pm (Biomarker Evaluation & Supervision Team for Personalized Medicine), in the field of medical oncology, radiation oncology, surgery, pathology, radiology, genetics, and medical physics in Imam Khomeini Hospital Complex, Tehran University of Medical Sciences have established and launched this center. We have several aims for diagnosis and treatment in cancer patients and one of them is using personalized medicine in end-stage patients. this team had much success in the project and this case report is one of them. ME is a researcher in the team and he wrote the manuscript with support from RSHi who is a Prof. of Genetic Medicine that the genetic profile of the patient is checked by him. ZT proved this manuscript and supervised radiation for this patient. BA is an oncologist who supervised the total of this project in collaboration with NP. SE and RSHa a were involved in the diagnosis and treatment of patients and MO was monitoring all processes of function as the head of the team. All authors contributed to the article and approved the submitted version.

## Acknowledgments

The authors would like to thank Dr. Farideh Mehrani and Ms. Masoumeh Nezami for their excellent technical contribution to this manuscript.

## Conflict of interest

The authors declare that the research was conducted in the absence of any commercial or financial relationships that could be construed as a potential conflict of interest.

## Publisher’s note

All claims expressed in this article are solely those of the authors and do not necessarily represent those of their affiliated organizations, or those of the publisher, the editors and the reviewers. Any product that may be evaluated in this article, or claim that may be made by its manufacturer, is not guaranteed or endorsed by the publisher.
